# BMI-based obesity classification misses children and adolescents with raised cardiometabolic risk due to increased adiposity

**DOI:** 10.1186/s12933-023-01972-8

**Published:** 2023-09-04

**Authors:** J. Karina Zapata, M. Cristina Azcona-Sanjulian, Victoria Catalán, Beatriz Ramírez, Camilo Silva, Amaia Rodríguez, Javier Escalada, Gema Frühbeck, Javier Gómez-Ambrosi

**Affiliations:** 1 Department of Endocrinology and Nutrition, Clínica Universidad de Navarra, Avda. Pío XII 36, Pamplona, 31008 Spain; 2 Paediatric Endocrinology Unit, Department of Paediatrics, Clínica Universidad de Navarra, Pamplona, Spain; 3 Instituto de Investigación Sanitaria de Navarra (IdiSNA), Pamplona, Spain; 4 Metabolic Research Laboratory, Clínica Universidad de Navarra, Irunlarrea 1, Pamplona, 31008 Spain; 5 Centro de Investigación Biomédica en Red-Fisiopatología de la Obesidad y Nutrición (CIBERobn), Instituto de Salud Carlos III, Pamplona, Spain

**Keywords:** Obesity, BMI, Adiposity, Children, Adolescents, Diagnosis, Cardiometabolic risk

## Abstract

**Objective:**

To assess how inaccurately the body mass index (BMI) is used to diagnose obesity compared to body fat percentage (BF%) measurement and to compare the cardiometabolic risk in children and adolescents with or without obesity according to BMI but with a similar BF%.

**Methods:**

A retrospective cross-sectional investigation was conducted including 553 (378 females/175 males) white children and adolescents aged 6–17 years, 197 with normal weight (NW), 144 with overweight (OW) and 212 with obesity (OB) according to BMI. In addition to BMI, BF% measured by air displacement plethysmography, as well as markers of cardiometabolic risk had been determined in the existing cohort.

**Results:**

We found that 7% of subjects considered as NW and 62% of children and adolescents classified as OW according to BMI presented a BF% within the obesity range. Children and adolescents without obesity by the BMI criterion but with obesity by BF% exhibited higher blood pressure and C-reactive protein (CRP) in boys, and higher blood pressure, glucose, uric acid, CRP and white blood cells count, as well as reduced HDL-cholesterol, in girls, similar to those with obesity by BMI and BF%. Importantly, both groups of subjects with obesity by BF% showed a similarly altered glucose homeostasis after an OGTT as compared to their NW counterparts.

**Conclusions:**

Results from the present study suggest increased cardiometabolic risk factors in children and adolescents without obesity according to BMI but with obesity based on BF%. Being aware of the difficulty in determining body composition in everyday clinical practice, our data show that its inclusion could yield clinically useful information both for the diagnosis and treatment of overweight and obesity.

**Supplementary Information:**

The online version contains supplementary material available at 10.1186/s12933-023-01972-8.

## Background

Obesity prevalence has increased dramatically worldwide in the last decades [1]. Average body mass index (BMI) and prevalence of obesity has risen worldwide also in children and adolescents in the period 1975 to 2016 [2]. The global age-standardized prevalence of obesity observed in children and adolescents between 5 and 19 years old increased from 0.7 to 5.6% for girls and 0.9–7.8% for boys [2]. Likewise, prevalence has increased markedly in school children in developed countries with a 23.8% of boys and 22.6% of girls classified as having overweight or obesity in 2013 [3]. The medical definition of obesity considers it as a state of increased adipose tissue, not body weight, of a magnitude that might have a negative impact on health, being strongly associated with elevated morbidity and also mortality [4, 5]. In this sense, severe pediatric obesity is associated with an increased prevalence of cardiometabolic risk factors [6]. Moreover, it has been evidenced that development of obesity in early ages predicts obesity [7] and cardiovascular events [8] in adulthood.

Despite obesity being defined as an excessive amount of adipose tissue, the most frequently used clinical tool to diagnose obesity in children, adolescents and adults is the BMI, since measurement of body fat is rarely available in routine clinical practice. However, absolute cutoffs cannot be established in children and adolescents given that BMI distribution varies markedly with growth [9]. It is recommended to diagnose a child (> 2 years) or adolescent as with overweight if the BMI is ≥ 85th percentile and < 95th percentile for age and sex, and as with obesity if the BMI is ≥ 95th percentile [10]. Alternatively, the IOTF proposed age- and sex-specific BMI cutoffs for children and adolescents corresponding to the widely used adult obesity cutoffs of 25 and 30 kg/m^2^, pooling international data [11]. BMI shows a high specificity but low sensitivity to detect excess body fat percentage (BF%) at all ages [12], failing to identify around 25% of children with excess adiposity [13]. Moreover, epidemiological studies analyzing the degree of misclassification using BMI to diagnose obesity in pediatric population as well as the impact of BF% on the levels of cardiometabolic risk factors in children and adolescents are scarce [14, 15]. Since BF% has been shown to determine cardiometabolic risk even in subjects with a BMI within the normal range [12, 16] at the same time as being related to all-cause mortality in adults [17], we hypothesized that already in early ages identifying the clinical use of BF% measurement in determining the cardiometabolic risk linked with obesity in pediatric patients may be crucial.

With this purpose, we used data from an existing cohort of a cross-sectional investigation with two objectives: first, to determine the degree of misclassification in obesity diagnosis with the use of BMI as compared to the direct measurement of BF% in children and adolescents. Second, to evaluate whether the cardiometabolic risk factor profile in subjects without obesity according to BMI but classified as with obesity by BF% cutoff points is different from that of subjects with obesity defined by BMI with a similar BF%.

## Methods

### Study design and participants

A retrospective cross-sectional analysis was performed with 553 (378 females/175 males) white children and adolescents, aged 6–17 years, recruited for previous studies between January 2001 to February 2019 at the Pediatric Endocrinology Unit and the Dpt. of Endocrinology & Nutrition from the Clínica Universidad de Navarra (Pamplona, Spain), either for an annual routine health check-up (healthy children without long-term diseases) or consulting due to excess weight as previously described [18–20]. Children and adolescents were classified on the basis of age- and sex-specific BMI cutoffs as defined by Cole et al. [11]. Pubertal status was evaluated by a pediatrician and determined using the Tanner stage [21]. None of the participants was taking medication potentially affecting body composition.

A subset (subcohort 1) of 360 subjects (232 females/128 males) was considered in the subanalysis used to compare the cardiometabolic risk between individuals classified by BMI as without obesity and with obesity, but with a similar BF%. The analysis included as the group of reference the subjects classified as normal weight (NW) by both the Cole classification and BF% (Normal, 119 girls and 35 boys), and compared it to those classified as without obesity (with NW or overweight) by Cole but with obesity by BF% (NOOB, 52 girls and 39 boys) as well as to a BF%-matched group of subjects classified as having obesity by both Cole and BF% (OBOB, 61 girls and 54 boys). We followed a similar design as the one previously performed in adults [12], but using Cole’s classification instead as the usual BMI cutoff points used for adults.

In order to explore the effect of body composition on glucose metabolism, glucose and insulin curves were determined during an oral glucose tolerance test (OGTT) in a representative subgroup (n = 58, 5 Normal, 17 NOOB and 36 OBOB, subcohort 2) in whom OGTT was available. The study followed the ethical standards of the Declaration of Helsinki and the experimental protocol was approved by the Research Ethical Committee of the *Universidad de Navarra* (protocol 2020.236). Signed informed consent was obtained from the parents or legal guardians and from the participants over 12 years old. Children under 12 years assented to participate.

### Anthropometric measurements

The body composition and the anthropometric determinations as well as the blood sampling were performed on the same day. Height was measured with a Holtain stadiometer (Holtain, Crymych, UK), while body weight was determined with the air displacement plethysmography (ADP) calibrated electronic scale with individuals wearing a swimming suit. BMI was calculated as dividing weight in kg by the square of height in m. Waist circumference was determined at the midpoint placed between the iliac crest and the rib cage on the midaxillary line. Hip circumference was determined at the position yielding the maximum circumference over the buttocks. Blood pressure was measured after a 5-minute rest with a sphygmomanometer. Blood pressure was determined 3 times at the right upper arm and the average was used in the analyses.

### Body composition

Body density was calculated by ADP (Bod-Pod^®^, COSMED, Albano Laziale, Italy). Data for calculation of body fat by this plethysmographic technique has been published to agree closely with the classic gold standard underwater weighing (hydrodensitometry) in children [20, 22] and has been used as the reference method to validate other body composition measurements in children [23]. The Siri equation was used to calculate BF% from body density. Cutoff points for BF% utilized for defining overweight (20.1–24.9% for boys and 30.1–34.9% for girls) and obesity (≥25.0% for boys and ≥35.0% for girls) were similar to those most frequently used in the literature [14, 24–26].

### Blood biochemistry

Plasma and serum samples were obtained in the morning after an overnight fast to avoid potential confounding bias due to hormonal rhythms. Plasma glucose was measured by an automated analyzer (Modular P800, Roche, Basel, Switzerland) as previously reported [20, 27]. Insulin was quantified using an enzyme-amplified chemiluminescence assay (Immulite®, Diagnostic Products, Los Angeles, CA, USA). The homeostatic model assessment (HOMA) and the quantitative insulin sensitivity check index (QUICKI) were used as proxies of insulin resistance and insulin sensitivity, respectively. Triglyceride and total cholesterol concentrations were measured by enzymatic spectrophotometric methods (Roche). HDL-cholesterol was determined by a colorimetric method in a Beckman Synchron® CX analyzer (Beckman Instruments, Bucks, UK). LDL-cholesterol was estimated by the Friedewald formula. High-sensitivity C-reactive protein (CRP) was determined by the Tina-quant® CRP (Latex) ultrasensitive assay (Roche, Basel, Switzerland). Number of white blood cells (WBC) was studied using an automated cell counter (Beckman Coulter, Fullerton, CA, USA). Uric acid, alanine aminotransferase (ALT), aspartate aminotransferase (AST), γ-glutamyltransferase (γ-GT), and creatinine were determined by enzymatic tests (Roche). Leptin was quantified by a RIA method (Linco Research, St. Charles, MO, USA); intra-and inter-assay coefficients of variation were 5.0% and 4.5%, respectively.

### Statistical analysis

Data are presented as mean ± standard deviation (SD). Differences between groups were analyzed by ANOVA followed by Scheffé’s tests or Fisher’s Least Significant Differences, as appropriate. Log transformed CRP concentrations were used given their non-normal distribution. The remaining variables followed an adequate distribution for the application of parametric tests. Correlations between two variables were determined by Pearson (*r*) correlation coefficient. The calculations were performed by SPSS version 23 (Chicago, IL, USA) and GraphPad Prism 8 (La Jolla, CA, USA). *P* values lower than 0.05 were considered statistically significant.

## Results

### Baseline characteristics of the children and adolescents

The study population included 197 children and adolescents with NW, 144 with overweight and 212 with obesity according to Cole’s BMI-based classification (Table 1). Boys exhibited higher weight, BMI, waist-to-hip ratio (WHR), waist-to-height ratio (WHtR), visceral adiposity and blood pressure, while girls showed increased BF% as expected, as usually girls have a higher BF% than boys. Average Tanner stage was higher in girls, since they were slightly older (without significant differences in age) and puberty takes place before in girls (Table 1). Analyzing the whole cohort of children and adolescents, BMI and BF% showed a strong correlation (*r* = 0.79; *P* < 0.001), which following stratification by gender was stronger for females (*r* = 0.85; *P* < 0.001) and weaker for males (*r* = 0.75; *P* < 0.001) (Fig. 2A).

**Table 1:** Characteristics of children and adolescents included in the analysis according to sex

	Boys	Girls	*P*
n	175	378	
Age, y	13.7 ± 2.7	14.2 ± 2.9	0.057
Height, m	1.63 ± 0.15	1.59 ± 0.11	< 0.001
Weight, kg	70.8 ± 21.7	63.3 ± 19.1	< 0.001
BMI, kg/m^2^	26.3 ± 5.7	25.0 ± 6.3	0.022
Body fat, %	30.4 ± 13.2	33.2 ± 10.5	0.012
WC, cm	88 ± 15	81 ± 14	< 0.001
WHR	0.90 ± 0.07	0.83 ± 0.08	< 0.001
WHtR	0.55 ± 0.09	0.51 ± 0.09	< 0.001
Visceral fat (cm^2^)	124 ± 80	76 ± 38	< 0.001
Tanner stage	2.41 ± 1.41	3.40 ± 1.65	< 0.001
Stage 1, %	31	19	
Stage 2, %	35	19	
Stage 3, %	14	11	
Stage 4, %	3	5	
Stage 5, %	17	46	
SBP, mm Hg	110 ± 13	105 ± 12	< 0.001
DBP, mm Hg	66 ± 8	64 ± 8	0.016
Cole’s classification			
Normal weight, n (%)	47 (27)	150 (40)	
Overweight, n (%)	41 (23)	103 (27)	
Obesity, n (%)	87 (50)	125 (33)	

Data are mean ± SD or n (%). BMI, body mass index; WC, waist circumference; WHR, waist-to-hip ratio; WHtR, waist-to-height ratio; SBP, systolic blood pressure; DBP, diastolic blood pressure. Differences between sexes were analyzed by unpaired Student’s *t* tests

**Fig. 1:**
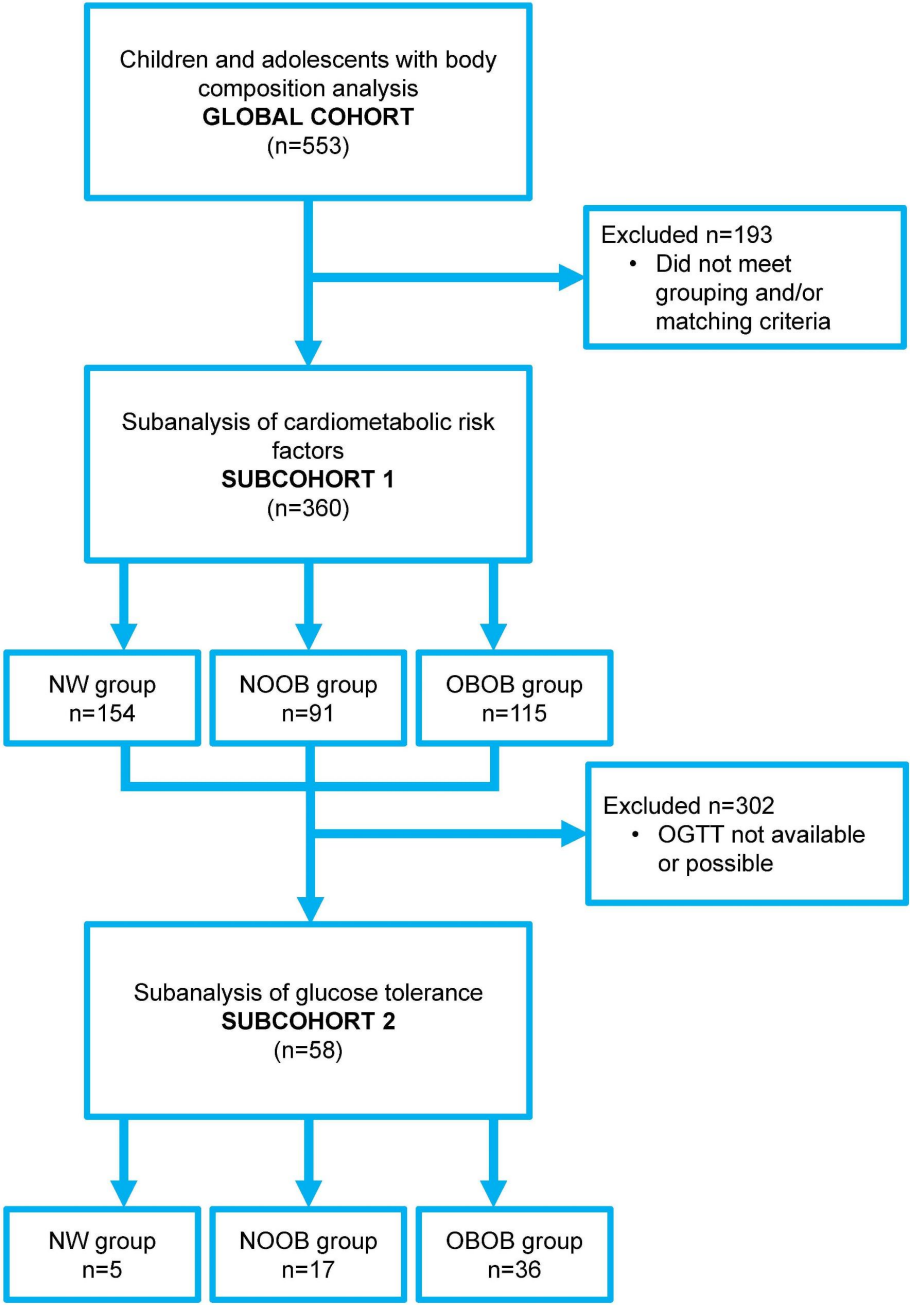
Flow chart of children and adolescents included in the global sample and in the different substudies. NW, normal weight by both the Cole classification and BF%; NOOB, individuals classified as without obesity (with NW or overweight) by Cole but with obesity by BF%; OBOB, BF%-matched group of subjects classified as having obesity by both Cole and BF%; OGTT, oral glucose tolerance test

**Fig. 2:**
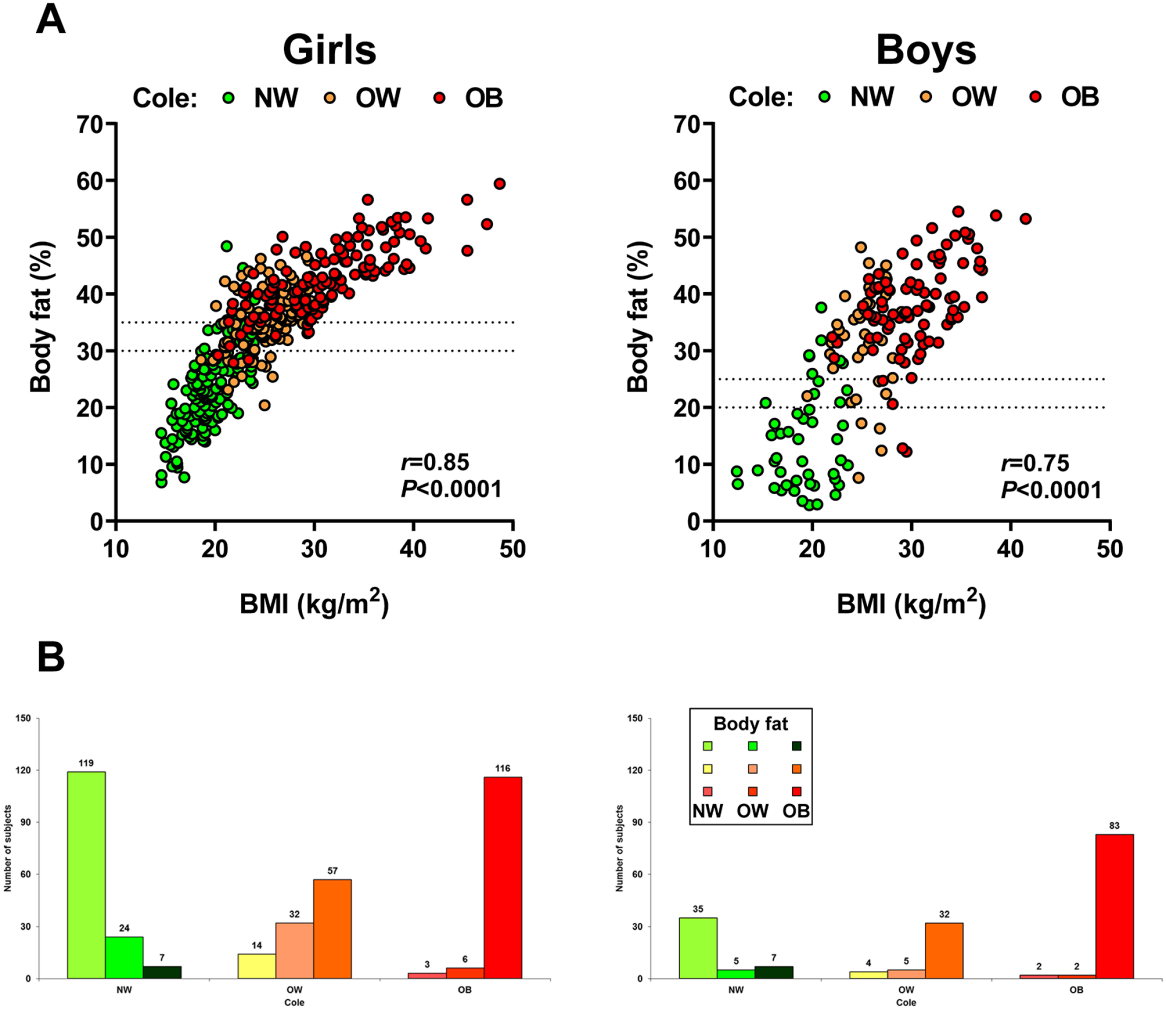
Association of body mass index and body fat percentage stratified by gender in children and adolescents. **A**) Left: girls (n = 378). Right: boys (n = 175). Horizontal lines delimit cutoffs for defining overweight and obesity according to BF% (30.0 and 35.0% in girls and 20.0 and 25.0% in boys, respectively). **B**) Classification of subjects as normal, with overweight or with obesity according to body mass index (Cole’s classification) or body fat percentage. Overweight was defined as BF% between 20.1–24.9% in boys or between 30.1–34.9% in girls, while obesity was defined as BF ≥25.0% in boys or ≥35.0% in girls

### Use of BMI misclassifies children and adolescents according to BF%

The classification of the subjects of the global cohort as with NW, overweight or obesity according to either Cole’s classification or BF% is depicted in Figs. 2B and Supplementary Fig.1. Depending on the criterion utilized, considerable discrepancies were found for classification. On the basis of the real BF% figures, 22% of the NW participants by the established BMI criteria were misclassified, exhibiting either overweight (15%) or obesity (7%) taking in account their BF%, while only 2% of subjects with obesity using BMI were NW according to BF%. Furthermore, 62% of those subjects classified as having overweight according to Cole had actually obesity considering their BF% (Fig. 2B and Supplementary Fig. 1). This misclassification was higher in boys (15% of those classified as NW by Cole’s had obesity by BF%, while 78% of those with overweight based on BMI had obesity by BF%), than in girls (5% of those classified as NW by Cole’s had obesity according to their BF% and 55% of girls classified as overweight according to BMI had obesity by BF%). The misclassification is also visible in the scatter-plots that compare BF% with BMI with the cutoff points indicated by reference lines for both girls and boys (Fig. 2A). In the global cohort, Cole’s classification had a specificity of 94% and a sensitivity of 66% to detect children and adolescents with obesity according to BF%.

**Fig. 3:**
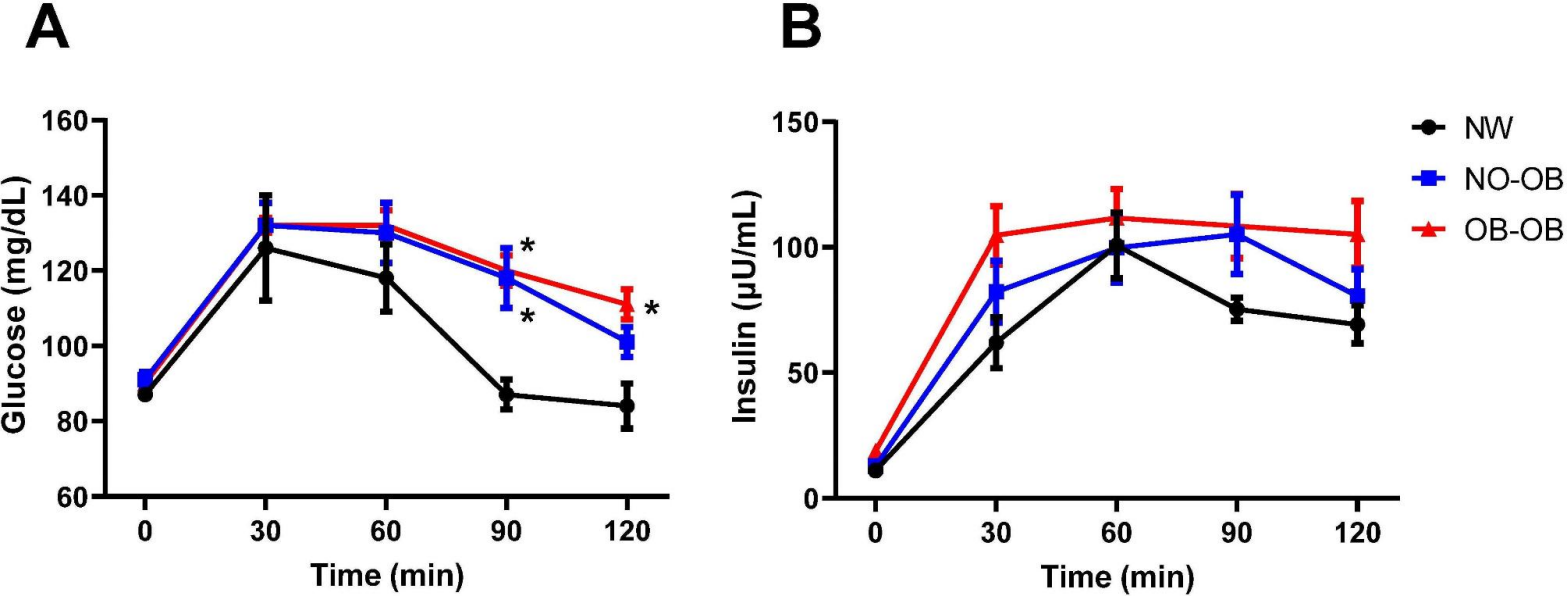
Glucose (**A**) and insulin (**B**) curves during an oral glucose tolerance test in 58 children and adolescents (subcohort 2) classified as normal weight by both Cole classification and body fat percentage (BF%) (Normal), as without obesity by Cole but with obesity by BF% (NOOB) or as having obesity by both Cole and BF% (OBOB). Data are mean ± SD. Differences between groups were computed by ANOVA followed by LSD tests. *P < 0.05 vs. Normal group

### Increased cardiometabolic risk factors in children and adolescents without obesity according to BMI but with obesity based on BF%

In order to analyze the impact of the detected incorrect classification on the potential underestimation of the cardiometabolic risk associated with obesity, several risk factors were compared in subjects with a similar BF% well within the obesity range but considered either as non-obese (NOOB) or obese (OBOB) by Cole’s classification (subcohort 1). According to these criteria of matching, although boys from the OBOB group exhibited a higher BMI (*P* < 0.001) than those of the NOOB group, both groups had similar BF% (BF% approx. 35%; *P* = 0.998), which was well within the obesity range (Table 2). Both groups exhibited higher waist circumference (*P* < 0.001) and WHtR (*P* < 0.001) than the NW group being higher in the OBOB group. In the groups with obesity according to BF% WHtR was > 0.50 suggesting increased cardiometabolic risk in those patients. Interestingly, no differences in both WHR (*P* = 0.527) and visceral fat (*P* = 0.244) were observed between the groups with obesity by BF%. Systolic blood pressure (SBP) was elevated in the NOOB group and further raised in the OBOB group (*P* = 0.035 vs. NOOB), while no differences were observed in diastolic blood pressure (DBP) (*P* = 0.097). Plasma glucose levels were similar between the three groups. However, insulin concentrations, HOMA and QUICKI were altered only in the OBOB group (*P* < 0.05, as compared to NOOB). Serum triglyceride levels were increased in the OBOB group as compared to the other groups (*P* < 0.001 and *P* < 0.05, as compared to the NW and NOOB groups, respectively). Total, LDL- and HDL-cholesterol serum levels were overall similar in the three groups. No differences in uric acid, ALT or the AST/ALT ratio were observed. However, γ-GT levels were increased only in the NOOB (*P* < 0.01 vs. NW). Interestingly, the marker of inflammation CRP was significantly higher in both groups with obesity (*P* < 0.05 and *P* < 0.01, NW vs. NOOB, and NW vs. OBOB, respectively) showing no differences between them (*P* = 0.670). As expected, leptin levels were significantly elevated in both groups with obesity (*P* = 0.002 vs. NW), exhibiting no differences between them (*P* = 0.957).

**Table 2:** Characteristics of subgroups of individuals according to Cole’s classification and body fat criteria in subcohort 1

	Boys				Girls			
	Normal (Cole and BF%)	Normal-OW Cole/Obesity BF% NOOB	Obesity-Cole/Obesity BF% OBOB		Normal (Cole and BF%)	Normal-OW Cole/Obesity BF% NOOB	Obesity-Cole/Obesity BF% OBOB	
	(*n* = 35)	(*n* = 39)	(*n* = 54)	*P*	(*n* = 119)	(*n* = 52)	(*n* = 61)	*P*
Age (years)	14.7 ± 2.2	13.4 ± 2.5	13.5 ± 2.5	0.064	15.3 ± 1.7	15.0 ± 2.4	14.7 ± 2.4	0.169
Weight (kg)	54 ± 14	62 ± 14	81 ± 22*†	< 0.001	50 ± 7	68 ± 11*	83 ± 15*†	< 0.001
BMI (kg/m^2^)	18.8 ± 2.9	24.6 ± 2.6*	29.5 ± 3.4*†	< 0.001	18.9 ± 2.3	26.3 ± 2.4*	31.9 ± 4.0*†	< 0.001
Body fat (%)	10.1 ± 5.0	35.0 ± 6.0*	35.1 ± 3.9*	< 0.001	21.0 ± 5.5	40.2 ± 3.3*	41.7 ± 3.5*	< 0.001
Waist (cm)	70 ± 7	85 ± 8*	96 ± 12*†	< 0.001	68 ± 6	86 ± 8*	96 ± 10*†	< 0.001
WHR	0.84 ± 0.05	0.91 ± 0.06*	0.92 ± 0.06*	< 0.001	0.77 ± 0.06	0.84 ± 0.06*	0.87 ± 0.08*†	< 0.001
WHtR	0.42 ± 0.04	0.54 ± 0.04*	0.59 ± 0.04*†	< 0.001	0.42 ± 0.04	0.54 ± 0.04*	0.60 ± 0.06*†	< 0.001
Visceral fat (cm^2^)	39 ± 15	138 ± 35*	163 ± 58*	< 0.001	36 ± 11	91 ± 18*	113 ± 34*†	< 0.001
Tanner stage	3.0 ± 1.6	2.1 ± 1.2	2.7 ± 1.5	0.275	4.0 ± 1.4	4.0 ± 1.4	3.9 ± 1.5	0.936
SBP (mm Hg)	102 ± 13	109 ± 11*	115 ± 10*†	< 0.001	98 ± 10	108 ± 11*	110 ± 10*	< 0.001
DBP (mm Hg)	60 ± 7	65 ± 6*	68 ± 6*	< 0.001	60 ± 8	66 ± 7*	67 ± 7*	< 0.001
Glucose (mg/dL)	87 ± 6	88 ± 8	89 ± 6	0.308	83 ± 6	87 ± 7*	88 ± 7*	0.001
Insulin (µU/mL)	5.8 ± 4.0	9.7 ± 6.2	18.0 ± 15.9*†	0.005	7.7 ± 4.4	13.0 ± 8.2	22.4 ± 19.4*†	< 0.001
HOMA	1.29 ± 0.92	2.13 ± 1.42	4.03 ± 3.62*†	0.005	1.66 ± 1.00	2.84 ± 1.90	4.87 ± 4.20*†	< 0.001
QUICKI	0.38 ± 0.04	0.36 ± 0.04	0.33 ± 0.04*†	< 0.001	0.37 ± 0.04	0.34 ± 0.04*	0.32 ± 0.04*†	< 0.001
Triglycerides (mg/dL)	53 ± 15	71 ± 21	91 ± 39*†	< 0.001	63 ± 20	70 ± 28	91 ± 48*†	< 0.001
Cholesterol (mg/dL)	159 ± 25	176 ± 47	166 ± 27	0.275	163 ± 28	156 ± 21	154 ± 30	0.177
LDL cholesterol (mg/dL)	96 ± 19	107 ± 42	98 ± 22	0.433	88 ± 24	91 ± 19	87 ± 26	0.703
HDL cholesterol (mg/dL)	53 ± 10	56 ± 17	50 ± 13	0.272	63 ± 14	51 ± 9*	49 ± 12*	< 0.001
Uric acid (mg/dL)	4.9 ± 1.7	5.4 ± 1.4	6.1 ± 0.8	0.130	3.9 ± 0.8	4.6 ± 0.8*	4.8 ± 0.8*	0.002
ALT (IU/L)	19 ± 17	27 ± 21	20 ± 9	0.277	14 ± 8	13 ± 6	16 ± 13	0.245
AST/ALT ratio	0.97 ± 0.24	1.06 ± 0.46	1.03 ± 0.47	0.860	1.23 ± 0.29	1.17 ± 0.40	1.07 ± 0.69	0.236
γ-GT (IU/L)	9 ± 3	18 ± 9*	14 ± 4	0.003	11 ± 9	11 ± 4	13 ± 4	0.431
CRP (mg/L)	0.7 ± 0.7	2.1 ± 2.1*	5.9 ± 10.6*	0.003	0.7 ± 0.3	2.8 ± 1.9*	2.0 ± 1.2*	< 0.001
WBC (10^6^ cells/mL)	5.4 ± 1.1	6.2 ± 1.5	5.8 ± 1.3	0.220	5.9 ± 1.8	7.2 ± 1.7*	7.2 ± 1.8*	< 0.001
Leptin (ng/mL)	4.5 ± 2.6	22.1 ±13.7*	21.1 ± 10.6*	< 0.001	10.5 ± 5.5	32.8 ±13.3*	43.8 ± 21.5 *	< 0.001

Data is presented as mean ± SD. Differences between groups were computed by ANOVA followed by Scheffé’s tests. * *P* < 0.05 vs. Normal. **†***P* < 0.05 vs. NOOB. CRP concentrations were log transformed for statistical analysis. ALT, alanine aminotransferase; AST, aspartate aminotransferase; BMI, body mass index; CRP, C-reactive protein; DBP, diastolic blood pressure; γ-GT, γ-glutamyltransferase; HOMA, homeostatic model assessment; QUICKI, quantitative insulin sensitivity check index; SBP, systolic blood pressure; WHR, waist-to-hip ratio; WHtR, waist-to-height ratio

In girls, BMI, waist circumference, WHR, WHtR and visceral fat were significantly increased in both groups with obesity, being higher in the OBOB in comparison to the NOOB group, while BF%, as the matching criterion, was similar in both groups with obesity according to adiposity (BF approx. 41%; *P* = 0.219). Comparable to what was shown in boys, CRP and leptin levels were increased in the NOOB and OBOB groups as compared to NW (*P* < 0.05) exhibiting no differences between them. Moreover, both groups of girls with obesity showed altered blood pressure, fasting glucose levels, HDL-cholesterol, uric acid and WBC as compared to NW (*P* < 0.01), but exhibiting no differences between them. Given that the pubertal state can decidedly influence body composition, data were analyzed according to Tanner stage. Noteworthy, no differences regarding the Tanner staging were observed, being similar between groups (Table 2 and Supplementary Table 1).

### Children and adolescents without obesity according to BMI but with obesity based on BF% exhibit altered glucose tolerance

In order to further explore the potential impact of the increased adiposity on glucose homeostasis we analyzed a subsample of the pediatric patients (n = 58) in whom OGTT was available (subcohort 2). Both groups of children and adolescents with obesity according to adiposity exhibited a higher area under the glucose curve as compared to the NW group (Fig. 3A). Significantly higher values of glucose levels were found in both groups with obesity 90 min after glucose intake (NW 87 ± 10, NOOB 118 ± 32 and OBOB 120 ± 24 mg/dL; *P* = 0.030), while 120 min after glucose intake, glucose levels were only significantly different in the OBOB group as compared to the NW one, being similar to that of the NOOB group (NW 84 ± 13, NOOB 101 ± 17 and OBOB 111 ± 25 mg/dL; *P* = 0.026). Insulin curves after glucose intake in the groups with obesity according to BF% were slightly higher than those of the NW group, but the differences were not statistically significant (Fig. 3B).

## Discussion

Obesity can be defined as an excessive accumulation of adipose tissue, and the degree of this excess is associated with the development of comorbidities [28–32]. We report herein that 7% of children and adolescents classified as having NW according to BMI, and 62% of those classified as overweight according to BMI had a BF% in the obesity range. On the other hand, only 2% and 4% of the children and adolescents with a BF% in the NW or overweight range, respectively, were misclassified as having obesity according to the BMI value. These results confirm that BMI, despite being a very practical and highly useful tool for epidemiological investigations, underestimates BF particularly in the overweight category, even at early ages. Published results showed an average sensitivity of 73–82% to detect severe adiposity and a mean specificity of 93–96% [13, 33]. This suggests that children and adolescents classified as having obesity by BMI (independently of the BMI-based criteria used) can almost definitely be considered as having obesity, while over a quarter of children not classified as having obesity by BMI might indeed have excess adiposity [13]. In the present study we found a similar 30.2% of children and adolescents classified as not having obesity according to BMI that actually exhibited a BF% within the obesity range, and an additional 14.7% of children pertaining to the NW group that had a BF% within the OW range. These findings indicate a notable degree of misclassification regarding the diagnosis of overweight and obesity in routine clinical practice using only BMI classifications, which translates in the underdiagnosis of children and adolescents at risk. This poses missed opportunities to identify patients with an increased cardiometabolic risk and to treat this life-threatening disease, similarly, although to a lesser degree, as it occurs in adults [12, 13, 34].

It has been shown that BF% and obesity elevates the risk of cardiovascular disease and T2D in adults [32, 35]. However, epidemiological studies which analyze the relation between BF% and blood levels of cardiometabolic risk markers in pediatric populations are scarce. Our study evidences for the first time that cardiometabolic risk factors are similarly altered in children and adolescents classified as with NW or overweight using BMI but with BF% within the obesity range that in those classified by BMI as having obesity with matched BF%. Data from the present study show that subjects without obesity by the BMI criterion but with obesity by BF% exhibit higher blood pressure and CRP in boys, and higher blood pressure, glucose, uric acid, CRP and WBC count, as well as reduced HDL, in girls, similarly to children and adolescents with obesity by both BMI and BF%. Importantly, both groups of school children with obesity by BF% showed altered glucose homeostasis in the OGTT as compared to their NW counterparts. These observations point out that by using the BMI-based classification systems for the diagnosis of obesity without directly assessing the body composition a huge opportunity of detecting children and adolescents with increased cardiometabolic risk factors due to excess adiposity who in the following years may have more serious conditions is being lost. Additionally, our data offer a scientific justification for the observation that body composition underlies the better understanding of the cardiovascular risk in “normal-weight” individuals with elevated adiposity [36–38].

Studies analyzing the impact of BF% on cardiometabolic risk factors in children and adolescents are scarce and have been predominantly focused on comparing the influence of adipose tissue distribution rather than to study the effect of increased adiposity itself [39, 40]. In this sense, BF% shows lower [41], similar [38] or higher [42, 43] association with cardiometabolic risk factors than measures of central adiposity. The present study indicates that the real amount of body fat plays a major role in the elevated cardiometabolic risk in children and adolescents. In this regard, the utilization of BF% cutoff points for the diagnostic of obesity allows the detection of more children and adolescents with increased cardiometabolic risk than the mere application of the BMI-based classification criteria [34]. The importance of adiposity distribution should not be underestimated. In the present study, boys did not present differences in WHR and visceral fat between the two groups with high adiposity, a finding that was not observed in girls, who exhibited lower values than males. Larger studies will be needed to analyze the influence of the amount and distribution of adiposity on cardiometabolic risk in children and adolescents.

The present study highlights the importance of determining body composition for the diagnosis of obesity even at early ages. This observation is particularly important taking into account the pathophysiological implications that elevated adiposity exerts in the context of normal- or over-weight. Although BMI is frequently employed as a proxy measure of body fat, it does not provide information on body composition, as demonstrated herein. Therefore, the solution is not reducing or changing the BMI cutoff values, something that would reduce specificity [13], but analyze in more detail body composition and distribution [44]. This is particularly necessary in the era of precision medicine [45]. Our study provides proof that a similar unfavorable cardiometabolic risk factor profile currently exists in children and adolescents with a non-obese BMI but a highly elevated adiposity compared to their counterparts with a BMI-based diagnostic of obesity.

One potential limitation of the present study pertains to the generalizability to other pediatric populations. This investigation was conducted in white children and adolescents and would need to be confirmed in other populations in order to determine ethnic or race-specific differences as regards to BF% ranges for a given BMI and whether the significant associations with the cardiometabolic risk factors are maintained [46]. Another potential limitation may relate to using Cole’s BMI classification system while there are other BMI-based criteria for the diagnosis of obesity in children and adolescents [47, 48]. However, the misclassification is consistently observed using different criteria [13]. Moreover, it could be viewed also as a strength since Cole’s BMI classification is not subject to the existing epidemiological influences and regional differences of z-scores or percentiles.

## Conclusion

The utilization of BMI for the diagnosis of obesity in children and adolescents in routine clinical practice underestimates the actual prevalence of this life-threatening disease given its association with the increase of cardiometabolic risk factors. Our study indicates that a marked number of children and adolescents with obesity according to their BF% are at risk of being underdiagnosed, and, therefore, opportunities for the most appropriate treatment instauration and comorbidity evaluation are being missed. In this regard, acknowledging how challenging it is to determine body composition in routine clinical practice, our data evidence that the inclusion of body composition measurements together with the assessment of morbidity for the diagnosis and the instauration of the most adequate treatment of obesity would be advisable.

### Electronic supplementary material

Below is the link to the electronic supplementary material.

Supplementary Material 1

## Data Availability

The datasets used and/or analyzed during the current study are available from the corresponding author on reasonable request.
