# Supplementary material for: BMI-based obesity classification misses children and adolescents with raised cardiometabolic risk due to increased adiposity
Source: Cardiovasc Diabetol. 2023 Sep 4;22:240. doi: 10.1186/s12933-023-01972-8 (PMC10476300; doi:10.1186/s12933-023-01972-8)
Supplement: Supplementary file 1 — Supplementary Material 1 [file 12933_2023_1972_MOESM1_ESM.pdf]

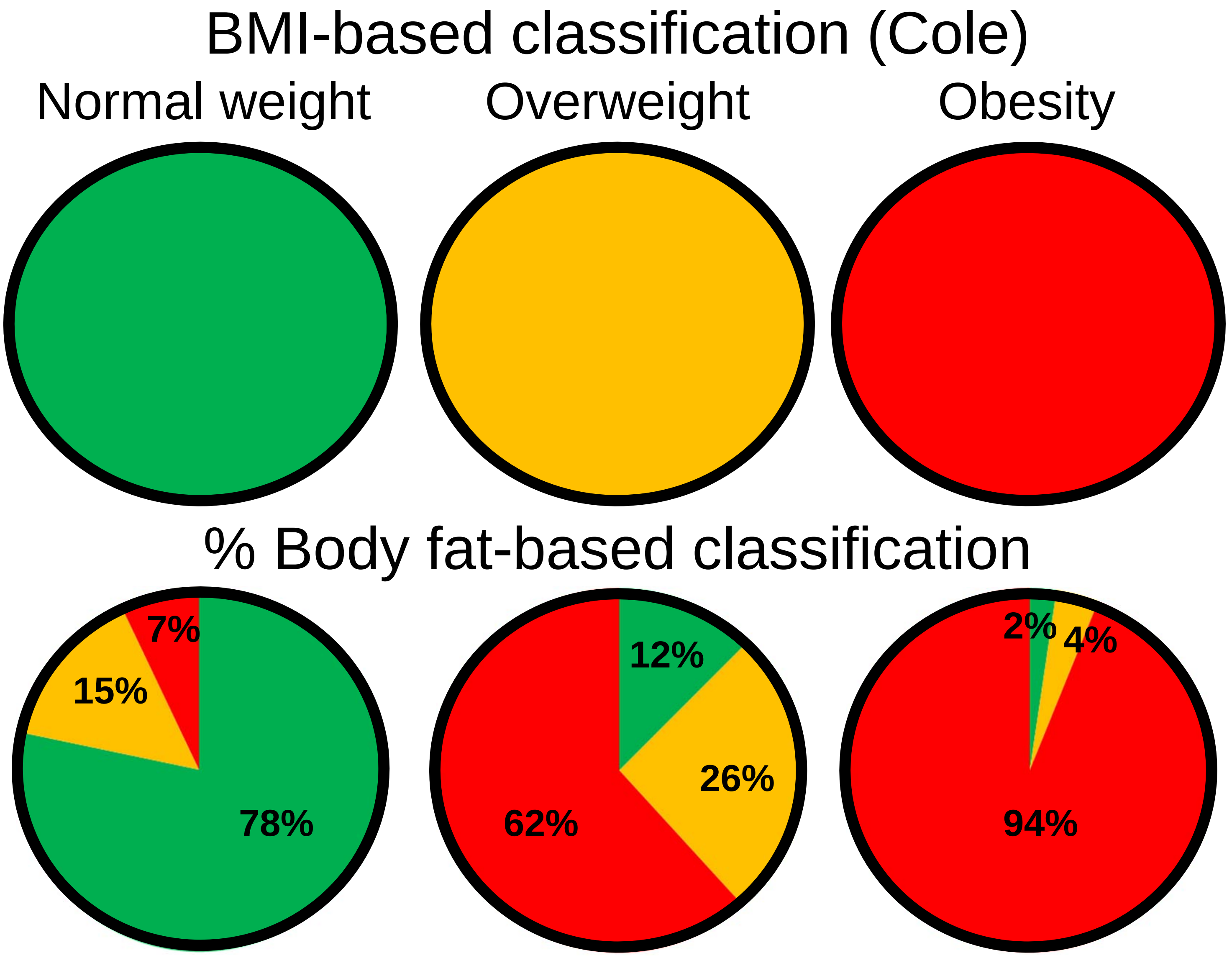

Supplementary Fig. 1 Percentages of misclassification according to body mass index (upper panel) and body fat percentage (lower panel) in children and adolescents of the global cohort (n=553) classified according to Cole.

Supplementary Table 1 Distribution of children and adolescents from subcohort 2 according to the established groups and Tanner stage

	Normal (Cole and BF%)	Normal-OW Cole/Obesity BF% NOOB	Obesity-Cole/Obesity BF% OBOB
Stage 1	15	35	50
Stage 2	14	44	42
Stage 3	24	29	47
Stage 4	50	17	33
Stage 5	28	31	41

Data are expressed as percentage. Differences in distribution were analyzed by Chi squared (*P*=0.564).
